# Pharmacogenomics of clinical response to Natalizumab in multiple sclerosis: a genome-wide multi-centric association study

**DOI:** 10.1007/s00415-024-12608-6

**Published:** 2024-09-12

**Authors:** Ferdinando Clarelli, Andrea Corona, Kimmo Pääkkönen, Melissa Sorosina, Alen Zollo, Fredrik Piehl, Tomas Olsson, Pernilla Stridh, Maja Jagodic, Bernhard Hemmer, Christiane Gasperi, Adil Harroud, Klementy Shchetynsky, Alessandra Mingione, Elisabetta Mascia, Kaalindi Misra, Antonino Giordano, Maria Laura Terzi Mazzieri, Alberto Priori, Janna Saarela, Ingrid Kockum, Massimo Filippi, Federica Esposito, Filippo Giovanni Martinelli Boneschi

**Affiliations:** 1 Laboratory of Human Genetics of Neurological Disorders, IRCCS San Raffaele Scientific Institute, Via Olgettina 60, Milan, Italy; 2 Laboratory of Precision Medicine of Neurological Diseases, Department of Health Science, University of Milan, Milan, Italy; 3 CRC “Aldo Ravelli” for Experimental Brain Therapeutics, Department of Health Science, University of Milan, Milan, Italy; 4 Institute for Molecular Medicine Finland (FIMM), University of FI Helsinki, Helsinki, Finland; 5 The Karolinska Neuroimmunology & Multiple Sclerosis Centre, Department of Clinical Neuroscience, Karolinska Institutet, Center for Molecular Medicine, Karolinska University Hospital, Visionsgatan 18, 171 76 Stockholm, Sweden; 6 Department of Neurology, School of Medicine, Technical University of Munich, Klinikum Rechts Der Isar, Ismaninger Str. 22, Munich, Germany; 7 Munich Cluster for Systems Neurology (SyNergy), Munich, Germany; 8 Department of Neurology and Neurosurgery, McGill University, Montréal, QC Canada; 9 Neurology Unit, IRCCS San Raffaele Scientific Institute, Via Olgettina 60, 20132 Milan, Italy; 10 Clinical Neurology Unit, Azienda Socio-Sanitaria Territoriale Santi Paolo E Carlo and Department of Health Sciences, University of Milan, Milan, Italy; 11 Neurorehabilitation Unit, IRCCS San Raffaele Scientific Institute, Via Olgettina 48, Milan, Italy; 12 Neurophysiology Service, IRCCS San Raffaele Scientific Institute, Via Olgettina 60, Milan, Italy; 13 Neuroimaging Research Unit, Division of Neuroscience, IRCCS San Raffaele Scientific Institute, Via Olgettina 60, Milan, Italy; 14 Vita-Salute San Raffaele University, Via Olgettina, 60, Milan, Italy

**Keywords:** Multiple sclerosis, Natalizumab, Pharmacogenomics, GRB2, LRP6

## Abstract

**Background:**

Inter-individual differences in treatment response are marked in multiple sclerosis (MS). This is true for Natalizumab (NTZ), to which a subset of patients displays sub-optimal treatment response. We conducted a multi-centric genome-wide association study (GWAS), with additional pathway and network analysis to identify genetic predictors of response to NTZ.

**Methods:**

MS patients from three different centers were included. Response to NTZ was dichotomized, nominating responders (R) relapse-free patients and non-responders (NR) all the others, over a follow-up of 4 years. Association analysis on ~ 4.7 M imputed autosomal common single-nucleotide polymorphisms (SNPs) was performed fitting logistic regression models, adjusted for baseline covariates, followed by meta-analysis at SNP and gene level. Finally, these signals were projected onto STRING interactome, to elicit modules and hub genes linked to response.

**Results:**

Overall, 1834 patients were included: 119 from Italy (R = 94, NR = 25), 81 from Germany (R = 61, NR = 20), and 1634 from Sweden (R = 1349, NR = 285). The top-associated variant was rs11132400_T_ (*p* = 1.33 × 10^–6^, OR = 0.58), affecting expression of several genes in the locus, like *KLKB1*. The interactome analysis implicated a module of 135 genes, with over-representation of terms like *canonical WNT signaling pathway* (*p*_adjust_ = 7.08 × 10^–6^). Response-associated genes like *GRB2* and *LRP6*, already implicated in MS pathogenesis*,* were topologically prioritized within the module.

**Conclusion:**

This GWAS, the largest pharmacogenomic study of response to NTZ, suggested MS-implicated genes and Wnt/β-catenin signaling pathway, an essential component for blood–brain barrier formation and maintenance, to be related to treatment response.

**Supplementary Information:**

The online version contains supplementary material available at 10.1007/s00415-024-12608-6.

## Introduction

Multiple Sclerosis (MS; MIM 126200) is a disease of the central nervous system (CNS) characterized by chronic inflammation, demyelination, and axonal loss [1]. It is a complex multifactorial disorder, with both genetic and environmental components playing a role in disease susceptibility. Genome-wide association studies (GWAS) greatly helped to elucidate genetic susceptibility for MS, revealing a highly polygenic architecture, with an ever-increasing number of common SNPs associated with risk [2].

Despite the expanded availability of multiple disease-modifying therapies (DMT), to date, no single drug has proven to be effective in controlling or delaying disease progression in the vast majority of patients. Further, the disease is highly heterogeneous and unpredictable in its expression and marked inter-individual differences have been observed in response to different treatments: currently, there is in fact paucity of biological markers that can help identifying responders and non-responders before starting a new drug [3, 4]. The identification of such biomarkers can help the neurologist to optimize treatment strategies, thus performing treatment decisions in clinical practice on an individual or stratum basis.

This is a critical need also for Natalizumab (NTZ), a second-line DMT approved in 2004 for relapsing–remitting course of MS, with established clinical efficacy in reducing the rate of clinical relapses, risk of sustained disability progression, and the number of new or enlarging brain lesions on Magnetic Resonance Imaging (MRI) [5, 6].

The drug is a humanized monoclonal antibody that selectively inhibits α4β1 and α4β7 integrins, expressed on the surface of lymphocytes, hindering their binding to vascular endothelial adhesion molecules and their migration in the CNS across the blood–brain barrier (BBB), with the result of diminishing inflammation [7].

Despite the high efficacy, a subset of patients treated with NTZ, estimated around 25% [8], do not respond or respond sub-optimally to the drug. To date, only a few candidate gene studies [9, 10] have been pursued to identify the factors that can genetically influence the response to NTZ, mostly relying on the putative mechanism of action of the drug. The difficulty in collecting large cohorts of well-phenotyped patients has hampered sufficiently powered pharmacogenetic studies.

In this multi-center study, we report the results from a meta-analysis of genome-wide screens of common variants with response to NTZ in cohorts of MS patients from Italy, Germany and Sweden followed up for 4 years. Analyses were conducted at variant and pathway level, followed by a network approach to investigate joint association signals and to facilitate elucidation of mechanisms underlying response to drug. We then tested association of two genes emerging from a previous candidate study of response to NTZ [9], focusing on detoxification enzymes that counteract toxic compounds of oxidative stress (OS): a variety of reactive oxygen species are in fact produced in MS pathogenesis, enhancing mitochondrial injury, energy failure, and consequent oligodendrocyte apoptosis[11] and putative role in response may be played by detoxification enzymes in the context of OS.

## Patients and methods

### Study population

The study included patients enrolled at three centers, from Italy, Germany, and Sweden.

Given the diversity in clinical data collection, a harmonization effort was put in place using data dictionary, which defined the variables that were then utilized in the common harmonized database. Data dictionary also defined the unit types that the data would be transformed to, and the final values for enumeration type variables. The data from the three centers were then mapped into the data dictionary variables by providing file name, column name, and unit type for each, and in case of enumeration types, the values were mapped to the harmonized values.

For each center, we constructed the study cohort including patients for which there were imputed data and complete availability of baseline variables like age and disease duration and the number of relapses 2 years before starting NTZ.

We excluded: (i) patients with age at treatment start <18 years and >55 years, (ii) patients who were on progressive courses (primary or secondary) at treatment start, and (iii) patients with Expanded Disability Status Scale (EDSS) >4 at treatment start, given that these patients were likely in secondary progressive phase and thus different from the rest of the cohort.

The resulting values from harmonization phase were generated in longitudinal format, where each row corresponded to one treatment exposure for the patient. If a patient had multiple exposures to NTZ, the first observation with exposure >12 months was considered eligible. If none of the observations had exposure larger than this threshold, the observation with the largest exposure was considered independently of the order.

In case of a previous short exposure to NTZ, the patient was included in the analysis only if the interval between the two exposures was longer than 12 months, to avoid any reactivation/rebound activity during the second NTZ treatment related to the withdrawal of the previous exposure. In the same way, patients previously treated with fingolimod were not included in the analysis, unless the interval between fingolimod withdrawal and NTZ start was longer than 12 months.

The study was approved by the local ethical committees.

### Response to therapy

We assessed response to NTZ with a dichotomous outcome, designating as responders patients who were relapse-free in the 4 years follow-up and non-responders those who experienced at least one relapse. Relapses were defined as new symptoms or exacerbation of existing symptoms persisting for ≥24 h, in the absence of concurrent illness/fever, and occurring ≥30 days after a previous relapse.

### Quality control

Our study cohort was derived as a subset of a larger multi-centric dataset, that constituted the replication cohort for study on MS severity, performed in the context of the International Multiple Sclerosis Genetic Consortium (IMSGC). Details on pre-imputation quality control, phasing and imputation steps are thus described therein [12].

Quality control steps on imputed data were performed within data from each center. For the two centers that had multiple distinct genotyping platforms (Italy and Sweden), we performed post-imputation quality control after obtaining a unique merged dataset. On a ‘per-marker’ basis, we excluded variants that: (1) had a call rate less than 95%; (2) had minor allele frequency (MAF) below 5%; (3) deviated from Hardy–Weinberg Equilibrium exact test at *p* < 10^-5^. On a ‘per-individual’ basis we excluded subjects who had high rates of genotype missingness (>5%) and one member of each pair of samples that showed, across platforms and within centers, high degree of recent shared ancestry (up to the second degree of kinship) inferred by robust estimation of their kinship coefficient [13]. We finally used Principal Component Analysis (PCA) pruning from the data variants with a call rate less than 99% and regions of extended linkage disequilibrium, to control for population stratification and to discard individuals with outlying values in ancestry. We considered as outliers those samples being more than 4 standard deviations away from the mean of the first two PCs. All the quality control steps were performed with PLINK 2.0 [14].

### Association analysis

**Fig. 1:**
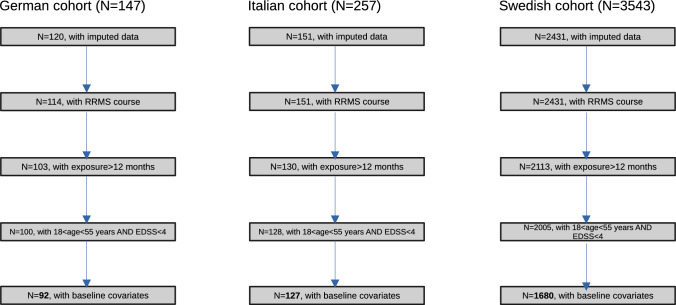
Patients’ workflow. For each participating center, the number of patients for each subsequent application of filtering criteria is reported. The sample size for final study cohort is reported in bold character

The workflow of the study is depicted in Fig. 1. We performed single-SNP association analysis fitting logistic regression models as implemented in PLINK 2.0, assuming additive effects of imputed continuous dosages of minor alleles. Models were adjusted for age and disease duration at treatment start, sex, the number of relapses in the 2 years preceding NTZ therapy, and the first five eigenvectors from PCA to account for population substructure. Summary statistics were aggregated using fixed-effect meta-analysis with inverse-variance weighting of log(odds-ratios), as implemented in PLINK 2.0.

Variants were annotated with ANNOVAR [15] and visualization of the top-associated locus was generated via regional plot with LocusZoom [16].

Gene-based analysis was conducted by means of Multi-marker Analysis of GenoMic Annotation (MAGMA, [17]) method v1.10, adjusting for the same set of covariates. The tool accounts for linkage disequilibrium and confounders like gene size and density. We used the *multi* option, which combines evidence from three models (principal components regression, mean of SNP squared Z-scores, and top SNP association).

A critical choice in gene-based and gene-set analysis is the assignment of SNPs to genes, since inclusion of noisy variants can be detrimental to association analysis. We assigned SNPs to the target gene with a “proximity rule” using a flanking window of 5 kb, to minimize overlap between nearby genes. Second, we applied a “functional rule” by:

- integrating in target genes cis-eQTL SNPs, based on significant SNP–gene associations in immune cells (FDR < 5%), as available in DICE repository [18], that identified common genetic variants that are associated with the expression of > 12,000 genes in 13 human immune cell types.
- integrating in target genes those variants significantly affecting splicing regulation (FDR<5%), using a catalogue of cis-splicing QTLs (sQTL, [19]) processed on transcriptome data in blood tissue from the Genotype-Tissue Expression Consortium [20].

Gene-wise statistics were then meta-analysed with weighted Stouffer’s procedure, which combines the Z-scores for each strata with weights set to the square root of the sample size. Approximately independent signals were identified upon application of a clumping procedure (primary p1 < 5 × 10^-5^, secondary *p*2 < 0.01, *r*^2^ > 0.6, maximum distance = 250 kb).

### Gene-set analysis

We used the gene-wise meta-analytic p values as input for gene-set analysis of association using as reference Gene Ontology (GO) Biological Processes, retrieved from Human Molecular Signature Database (MSigDB v2023.1, [21]). We filtered out GO terms with less than 10 and more than 500 annotated genes, finally testing 5376 gene-sets.

Each GO term was tested under the competitive null hypothesis, which states that aggregated variation in genes annotated to the gene set is no more associated with the outcome than that in all other genes in the genome. To accomplish this, we used as background signal the whole set of genes used in meta-analysis (*n* = 24,110).

### Network analysis

We performed a subnetwork detection analysis, projecting meta-analysed statistics onto STRING v11.5 reference interactome [22]. We retained only those links with high interaction evidence (score > 0.7) in one of the three evidence domains: (i) protein–protein interaction, derived from multiple interactomes, such as IntAct, BioGrid, MINT, and others; (ii) co-expression, which leverages gene expression data from multiple sources; (iii) databases, which collects evidence of interaction from curated pathway resources.

We then used dmGWAS tool [23], which applies a greedy search algorithm of dense modules within the node-weighted interactome, to detect association signals that aggregate in subnetworks. This procedure scores each module by a Z-score corresponding to the association level of the gene: the module score is obtained dividing the sum of the nodes scores by the square root of each module size. Starting from each seed, the procedure examines first-order neighbours and identifies those that generate the maximum increment of module score. We selected the top 1% of the top-scoring modules and merged them in a final subnetwork.

The top-scoring subnetwork was imported into Cytoscape v3.8 environment [24] for visualization, manipulation, and extraction of topologically relevant nodes (hubs, bottlenecks) with CentiScaPe plugin [25]. We computed distributions of graph centrality metrics like degree, betweenness and eigenvector centrality for the detected module and selected nodes residing in the top 5% of at least one of the four distributions: these metrics should measure the functional importance of genes in the module.

ClusterProfiler R package [26] was used to perform gene-set over-representation analysis with hypergeometric test of the genes annotated to the extracted module and the detected communities, using GO Biological Process domain as reference database; the list of genes from meta-analysis that were present on the filtered STRING interactome was used as background universe. Benjamini–Hochberg adjusted *p* values < 0.05 were used to nominate significant gene-sets.

## Results

### Association analysis

Clinico-demographic variables are reported for the three cohorts of the study population in Table 1. It can be observed that the sample size of the Swedish cohort (SWE) was much larger (*N* = 1634) as compared to the other two cohorts [*N* = 119 and *N* = 81 from Italy (ITA) and Germany (GER), respectively]. While there was some degree of heterogeneity at baseline variables, the proportion of R/NR did not significantly differ across the three cohorts (Table 1).

**Table 1:** Clinical and demographic characteristics of the three study cohorts

	Germany	Italy	Sweden	*P* value
	(*n* = 81)	(*n* = 119)	(*n* = 1634)	
Sex				ns*
Female	53 (65.4%)	81 (68.1%)	1,195 (73.1%)	
Male	28 (34.6%)	38 (31.9%)	439 (26.9%)	
Age drug start, years	33.4 (8.1)	34.2 (8.6)	36.7 (8.8)	< 0.001
Disease duration drug start, years	5.7 (6.1)	8.9 (5.9)	7.4 (6.2)	0.003
EDSS drug start ^§^	1.5 (1.5)	2 (1.5)	NA	ns
Relapses 2 years before drug start	1.9 (1.4)	2.1 (1.3)	1.3 (1.3)	< 0.001
Time exposed to NTZ, months	35.6 (11.7)	30.7 (12.7)	37.3 (12.5)	< 0.001
Relapses at 4 years	0.6 (1.0)	0.6 (1.0)	0.4 (0.8)	0.011
Response to treatment				ns*
Non responders	20 (24.7%)	25 (21%)	285 (17.4%)	
Responders	61 (75.3%)	94 (79%)	1,349 (82.6%)	

Values are presented as mean (standard deviation), unless otherwise stated

^**§**^Median (Inter-quartile range)

*p* values refer to the comparison among the three cohorts (*Chi-squared test, otherwise one-way ANOVA)

After quality control, a total of ~ 4.7 M variants were retained for downstream association analyses (SWE: 4768680, ITA: 4612675, and GER: 4716021). Fixed-effect inverse-variance weighted meta-analysis of effect sizes across the three strata was finally performed on a set of 4747971 variants shared between at least two of the three cohorts. QQ plot reported in Supplementary Fig. 1 did not show effect of genomic inflation due to population substructure.

**Fig. 2:**
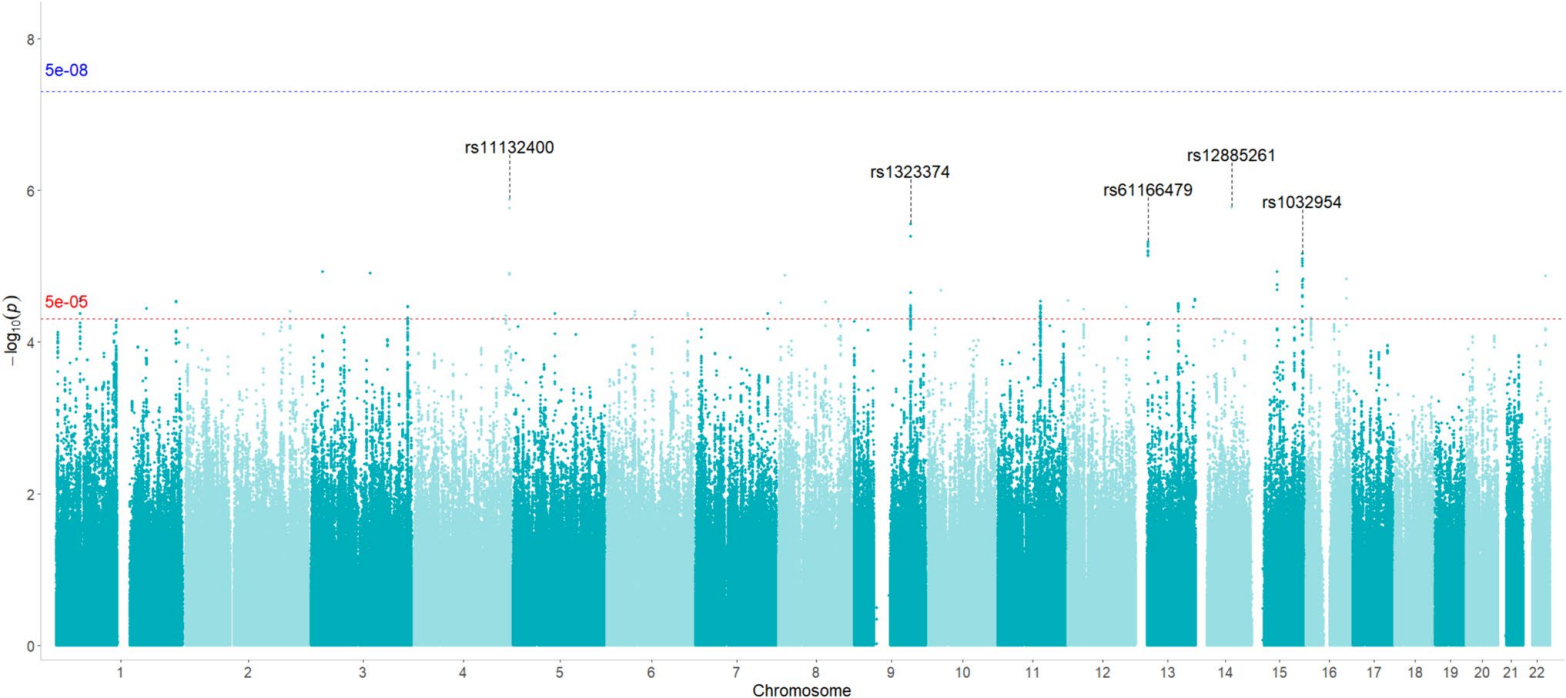
Manhattan plot. The Manhattan plot of − log_10_(*p*) of associations from fixed-effect meta-analysis. The genome-wide significance level is set at *p* = 5 × 10^–8^ (blue line), whereas suggestive significance threshold at *p* = 5 × 10^–5^ (red line). rsIDs of the top five associated SNPs are marked

**Fig. 3:**
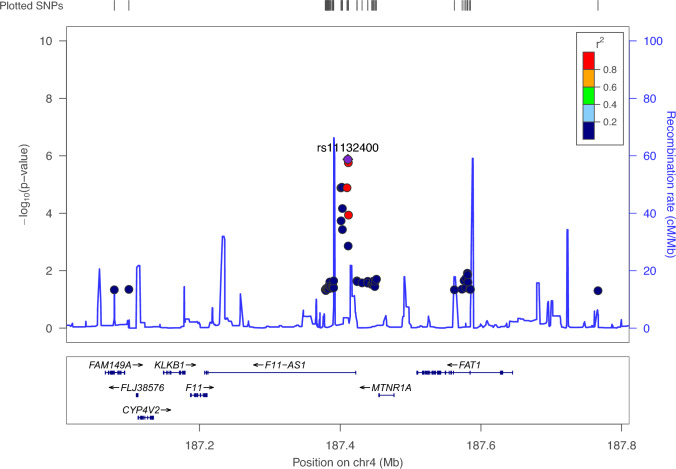
Regional plot of top-associated locus from fixed-effects meta-analysis. The plot shows the genomic context of associated signal (lead variant: rs11132400_T_, *p* = 1.33 × 10^–6^, OR = 0.58) mapping to intronic region of *F11-AS1* gene. The plot was generated with LocusZoom tool (http://locuszoom.sph.umich.edu). The − log_10_(p) of associations from fixed-effect meta-analysis is reported on left *y*-axis, and the recombination rate on right *y*-axis, over the genomic position (hg19). Each symbol represents one SNP, with the most associated SNP marked in purple and shading of the other points based on the linkage disequilibrium metrics with the top SNP. Positions of genes are shown below the plot

No variants showed significant association at genome-wide level (*p* < 5 × 10^−8^). Overall pattern of association is reported on the Manhattan plot in Fig. 2, whereas independent clumped variants associated at suggestive level (*p* < 5 × 10^–5^) are reported in Table 2. The most significant signal of association was detected at rs11132400_T_, located in the intronic region of the *F11-AS1* gene, coding for an antisense RNA, on chromosome 4 (*p* = 1.3 × 10^–6^, OR = 0.58, Fig. 3). For this variant, at least nominally significant eQTL effects in multiple tissues were detected using QTLbase [27]: In particular, eQTLs were identified in Induced Pluripotent Stem Cell for genes *KLKB1*, *CYP4V2*, *F11* and in Blood-Macrophage for *FAT1* gene*.*

**Table 2:** List of top-associated variants after meta-analysis. The list is obtained upon a clumping procedure (see *Methods* for details), filtered at *p* < 5 × 10^–5^. The effect allele is the minor allele

Position	Minor allele	Major allele	MAF	P	OR	SE	*I*^2^	Gene	Genomic context	Distance to gene
187,410,850	T	C	0.268	1.328E−06	0.582	0.112	0	*F11-AS1*	ncRNA_intronic	
68,071,570	T	C	0.421	1.678E−06	1.538	0.090	0	*PIGH; ARG2*	Intergenic	Dist = 4552; Dist = 15,067
111,037,829	T	A	0.251	2.793E−06	0.596	0.111	13	*KLF4; ACTL7B*	Intergenic	Dist = 785,779; Dist = 579,040
20,863,249	G	A	0.057	4.804E−06	2.122	0.165	0	*GJB6; CRYL1*	Intergenic	Dist = 56,715; Dist = 114,557
98,275,102	C	G	0.429	6.938E−06	0.650	0.096	34	*LOC101927310; LINC00923*	Intergenic	Dist = 171,174; Dist = 10,744
47,775,385	A	G	0.230	1.187E−05	1.561	0.102	0	*SEMA6D*	Intronic	
21,728,917	A	G	0.142	1.191E−05	0.510	0.154	0	*ZNF385D*	Intronic	
187,402,360	A	G	0.480	1.233E−05	1.496	0.092	0	*F11-AS1*	ncRNA_intronic	
115,392,100	A	G	0.347	1.259E−05	1.476	0.089	0	*GAP43*	Intronic	
12,783,519	T	C	0.075	1.325E−05	1.934	0.151	0	*LINC00681; TRMT9B*	Intergenic	Dist = 107,719; Dist = 19,663
43,181,587	T	C	0.080	1.362E−05	1.918	0.150	0	*A4GALT; ARFGAP3*	Intergenic	Dist = 64,280; Dist = 10,922
79,303,163	G	A	0.430	1.475E−05	1.469	0.089	0	*WWOX; MAF*	Intergenic	Dist = 56,599; Dist = 324,572
98,347,870	C	G	0.429	1.494E−05	0.676	0.091	57	*LINC00923*	ncRNA_intronic	
27,204,531	C	T	0.095	2.087E−05	1.775	0.135	42	*ABI1; FAM238C*	Intergenic	Dist = 54,515; Dist = 15,604
47,746,678	C	G	0.075	2.692E−05	1.839	0.145	29	*STIL*	Exonic	
113,293,608	G	A	0.411	2.744E−05	1.461	0.090	0	*TUBGCP3; ATP11AUN*	Intergenic	Dist = 51,109; Dist = 7750
606,173	C	A	0.143	2.862E−05	1.646	0.119	0	*B4GALNT3*	Intronic	
83,766,280	A	C	0.114	2.909E−05	1.739	0.132	48	*DLG2*	Intronic	
236,647,736	T	C	0.233	2.939E−05	1.495	0.096	0	*EDARADD*	UTR3	NM_145861:c.*1787C>T; NM_080738:c.*1787C>T
93,450,212	A	G	0.295	3.023E−05	1.471	0.093	0	*RUNX1T1; LOC102724710*	Intergenic	Dist = 334,599; Dist = 127,459
4,314,917	A	G	0.220	3.087E−05	1.523	0.101	0	*CSMD1*	Intronic	
81,279,746	A	G	0.271	3.116E−05	1.479	0.094	6.4	*SPRY2; LINC00377*	Intergenic	Dist = 364,485; Dist = 312,780
111,023,899	T	C	0.404	3.369E−05	1.441	0.088	0	*KLF4; ACTL7B*	Intergenic	Dist = 771,849; Dist = 592,970
189,243,943	T	C	0.413	3.424E−05	1.433	0.087	0	*TPRG1; TP63*	Intergenic	Dist = 200,850; Dist = 70,592
114,813,313	C	T	0.267	3.459E−05	1.483	0.095	0	*TBX5*	Intronic	
110,446,839	G	A	0.413	3.508E−05	1.433	0.087	0	*IRS2; LINC00396*	Intergenic	Dist = 7909; Dist = 258,793
177,987,660	G	A	0.137	3.639E−05	1.617	0.116	0	*CRYZL2P*	ncRNA_intronic	
31,271,788	A	G	0.072	3.733E−05	1.845	0.148	60	*OVOS2*	ncRNA_intronic	
206,468,850	T	G	0.419	4.007E−05	1.433	0.088	0	*PARD3B*	Intronic	
54,692,961	G	A	0.116	4.025E−05	1.652	0.122	6.9	*TINAG; FAM83B*	Intergenic	Dist = 438,021; Dist = 18,608
141,938,633	C	T	0.069	4.217E−05	1.890	0.155	74	*MGAM2; MOXD2P*	Intergenic	Dist = 16,509; Dist = 1923
81,959,114	T	C	0.499	4.222E−05	0.697	0.088	5.8	*ATP6AP1L; MIR3977*	Intergenic	Dist = 343,363; Dist = 176,860
158,058,545	T	C	0.436	4.257E−05	1.455	0.092	0	*ZDHHC14*	Intronic	
180,648,923	C	T	0.480	4.571E−05	0.699	0.088	0	*LINC01098; LINC00290*	Intergenic	Dist = 1,737,019; Dist = 1,336,320
83,182,354	A	G	0.299	4.605E−05	1.458	0.093	0	*DLG2*	Intronic	
10,732,844	G	A	0.050	4.841E−05	2.053	0.177	53	*TEKT5*	Intronic	
130,499,623	G	A	0.096	4.929E−05	1.749	0.138	42	*LINC01163; LINC02667*	Intergenic	Dist = 383,633; Dist = 211,474
156,675,608	A	C	0.135	4.934E−05	1.642	0.122	0	*GUCY1A1; GUCY1B1*	Intergenic	Dist = 17,399; Dist = 4565
50,679,081	T	G	0.372	4.966E−05	1.448	0.091	0	*DEFB112; TFAP2D*	Intergenic	Dist = 661,439; Dist = 2158

For each variant, the gene harboring it or nearest gene(s) are reported, together with its genomic context and distance in base-pairs from the nearest genes, as of ANNOVAR annotation

Abbreviations: MAF (Minor Allele Frequency), P (p value from fixed-effect meta-analysis), OR (odds ratio from fixed-effects meta-analysis), SE (standard error), I^2^ (heterogeneity index)

Other top-associated variants were rs12885261_T_, located in the intergenic region between genes *PIGH* and *ARG2* on chromosome 14 (*p* = 1.67 × 10^–6^, OR = 1.53) and rs1323374_T_, located in the intergenic region between genes *KLF4* and *ACTL7B* (*p* = 2.79 × 10^–6^, OR = 0.59) (Fig. 2). In QTLbase, rs12885261_T_ was found to exert eQTL effect in blood B cells on *ARG2* gene (T allele, beta = 0.31, *p* = 7.45 × 10^–8^).

The three mentioned variants exhibited an *I*^2^ heterogeneity index that was low (0% or 12.9%), reflecting concordance in effect sizes across the three cohorts, which can be observed in Supplementary Table 1, reporting the single-stratum effects of the SNPs in Table 2. We further report in Supplementary Table 2 the top ten variants with highest and lowest meta-analytic odds ratios.

Upon assignment of SNPs to genes according to proximity and function rules, a total of 24,110 autosomal genes had at least one assigned variant and 2,536,188 variants (63%) mapped for proximity, eQTL or sQTL effect to at least one gene. The complete list of meta-analysed genes from gene-based associations at *p* < 0.05 is reported in Supplementary Table 3.

In addition, we used gene-based statistics to test association for two genes, *NQO1* and *GSTP1* on chromosome 16 and 11, respectively, encoding for detoxification enzymes, whose nonsynonymous polymorphisms have been identified as associated with the response to NTZ in a candidate study [9]. Although we could not replicate findings at single-variant level for the two polymorphisms (rs1800566 in *NQO1* and rs1695 in *GSTP1*), our data from gene-based meta-analysis revealed nominal association for both genes (*p*_NQO1_ = 0.056, *p*_GSTP1_ = 0.029), indicating a possible role for them in the response to NTZ.

### Gene-set analysis

Gene-set analysis from meta-analysed genes under the competitive hypothesis did not yield significant results after multiple testing correction. Nevertheless, several GO terms that point to immune-related processes, in particular T helper cell differentiation, in response to NTZ were observed, like *Regulation of CD4 positive alpha beta T-cell differentiation* (*p* = 0.0009), *T helper 17 cell lineage commitment* (*p* = 0.0033749), *Positive regulation of adaptive immune response* (*p* = 0.00399), *Regulation of alpha beta T-cell differentiation* (*p* = 0.00435), *Negative regulation of type 2 immune response* (*p* = 0.0079), and *Regulation of T helper cell differentiation* (*p* = 0.00848). The complete set of nominally associated GO terms is reported in Supplementary Table 4.

### Network analysis

We searched for subnetworks with enriched genetic signals of response to NTZ, conducting dense module searching on the STRING high-confidence reference interactome, which consisted of 10,698 nodes, matched to meta-analysed genes, connected by 121,565 edges. The nodes were weighted by gene-based summary statistics from GWAS meta-analysis (*z*-score), aggregating the scores at the module level (see Methods). The algorithm identified 6,766 modules, and we prioritized those residing in the top-1% of the distribution of *z*-scores (*N* = 68), which exhibited extensive overlap. The minimum and maximum size of the top-ranking modules was 6 and 10 nodes, and the largest connected component obtained by merging them was a subnetwork of 135 nodes and 290 edges.

**Fig. 4:**
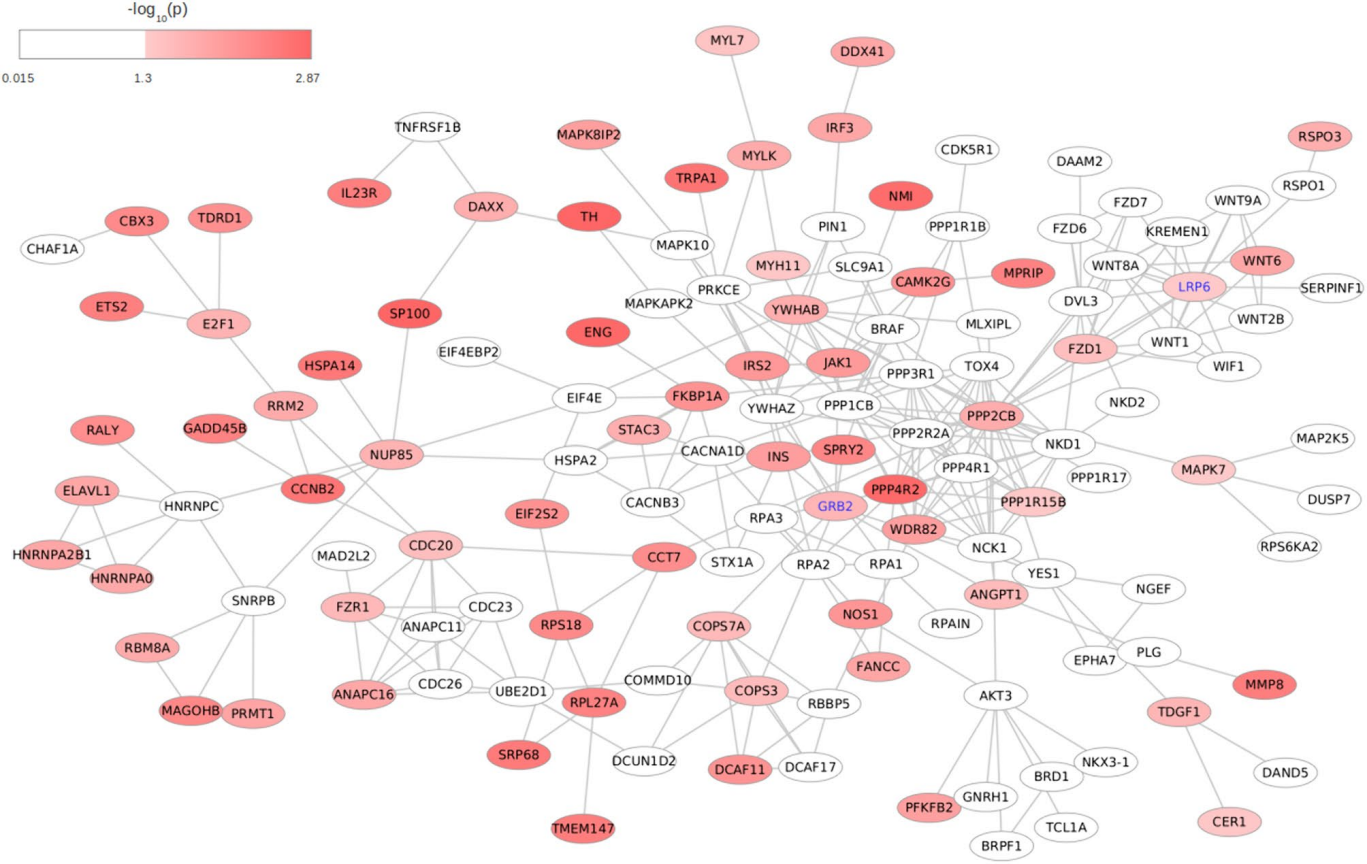
Detected network module. The final subnetwork resulting from the merge of modules, residing in top 1% of graph scores assigned by dmGWAS search algorithm, associated with response to NTZ. Color coding of nodes represents meta-analytic gene-level *p* values from MAGMA analysis, as indicated by the legend. Nodes/genes with association *p* > 0.05 were left white. Two genes which showed high level of centrality metrics and have already been implicated with MS (*GRB2* and *LRP6*) are highlighted

The top-associated genes in the module were *TH* (*p* = 1.31 × 10^–3^) and *SP100* (*p* = 1.33 × 10^–3^): by construction, not all genes which are part of the merged module were associated with response to NTZ: nevertheless, there were many of them which are directly or indirectly connected with associated genes (Fig. 4). From the detected module, we produced the most topologically important nodes prioritizing genes with values in the top 5% of the distribution of three node centrality metrics (Supplementary Table 5). The top-ranked genes like *PPP2CB* and *PPP4R2*, encode for part of protein complexes and thus shared high values of graph centrality metrics (degree, betweenness, and eigenvector centrality), Among other genes with topological relevance that were also significantly associated with response to NTZ, we identified two genes like *LRP6* (*p* = 0.045) and *GRB2* (*p* = 0.023) which have been already implicated in MS (see Discussion). Regional plots illustrating the overall pattern of association for the two genes are reported in Supplementary Fig. 2.

**Fig. 5:**
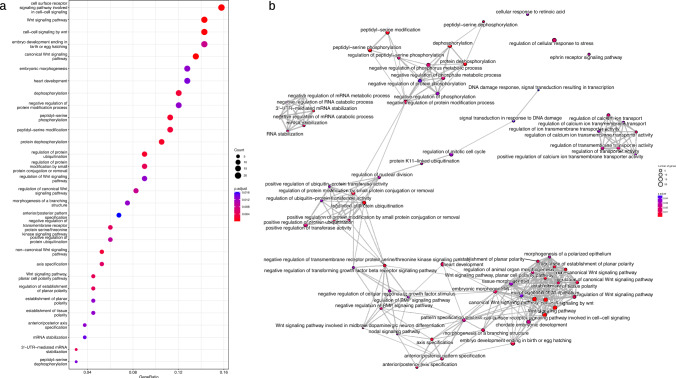
Enriched terms from over-representation analysis of genes in the detected network module against Gene Ontology Biological Process database. **a** Dotplot displaying the first 30 associated GO terms. *p* values were calculated from hypergeometric test, with adjustment for multiple testing with Benjamini–Hochberg procedure at FDR < 5%. The Count parameter in the legend illustrates the number of genes annotated to GO term and belonging to module. On *x*-axis, Gene Ratio reports the ratio between the number of genes in the module annotated to the term and the overall number of genes in the module (*N* = 135). **b** Enrichment map, reporting a graph-based representation of semantic similarity measures between GO terms enriched at FDR < 5% (*N* = 75, see Supplementary Table 3). Terms with high similarity tend to cluster together: the stronger the similarity, the shorter and thicker the edges. The color of nodes is coded according to p.adjust from hypergeometric test, as reported in the legend. Similarity between terms was computed with Jaccard correlation coefficient

Functional gene-set over-representation analysis was performed to yield possible biological mechanisms of module interacting genes. From GO BP terms, 75 terms were significant at FDR < 5% (Fig. 5a). Many of these terms were semantically related due to GO hierarchical structure. Among them, according to semantic similarity estimated with Jaccard similarity coefficient, four themes emerged (Fig. 5b): *Canonical WNT signaling pathways* (*p*_adjust_ = 7.08 × 10^–6^); *Protein dephosphorilation* (*p*_adjust_ = 1.42 × 10^–3^); *mRNA stabilization* (*p*_adjust_ = 0.0144); *Regulation of calcium ion transmembrane activity* (*p*_adjust_ = 0.0161). The complete list of over-represented GO BP terms is reported in Supplementary Table 6, together with the annotated module genes.

## Discussion

Identification of genetic markers, together with other biomarkers, that associate with response to DMTs is a crucial clinical need for MS patients’ stratification and their tailored management. In the case of a highly effective treatment such as Natalizumab, to date, only a few candidate gene studies have been performed to elicit such markers [9, 10], mainly due to reduced sample size caused by the relatively low number of non-responders to the drug.

Here, we conducted a multi-centric GWAS of response to NTZ, to our knowledge the largest in pharmacogenomics of this DMT, that we pursued at multiple analytical levels. Our study could not identify any locus at genome-wide significance: nevertheless, the top-associated SNP rs11132400_T_, an intronic variant in *F11-AS1* gene*,* was found to have eQTL effects on multiple genes with biological plausibility, such as *KLKB1*, *F11*, and *FAT1*.

The gene *KLKB1* encodes prekallikrein, a protein which modulates the integrity of BBB, whereas *F11* encodes the coagulation factor XI. Notably, both proteins can act as important mediators of the adaptive immune response during neuroinflammation. Specifically, in the contact activation pathway, three proenzymes in blood (plasma factor XII “FXII”, factor XI “FXI”, prekallikrein “PK”, and high-molecular-weight kininogen “HK”) bind to a surface and cause blood coagulation and inflammation by activating their respective enzymes (FXIIa, FXIa and α-kallikrein). Several lines of evidence show that *F11* and *KLKB1* are also implied in MS aetiology. Indeed, targeting of factor FXI improves neurological function and attenuates CNS damage in Experimental Autoimmune Encephalomyelitis (EAE), the animal model of MS [28]. Moreover, a deficiency of plasma prekallikrein, the precursor of kallikrein which is found to be upregulated in EAE, leads to decreased immune cell trafficking in the course of neuroinflammation rendering mice less susceptible to the disease [29, 30].

As of *FAT1* gene, its product functions as an adhesion molecule and as signaling receptor, and its importance in developmental processes and cell communication is well assessed. Several lines of evidence show that FAT1 activates a variety of signaling pathways through protein–protein interactions, including the Wnt/β-catenin and MAPK/ERK signaling pathways, which affect cell proliferation, migration, and invasion [31].

Interestingly, other intronic variants in *F11-AS1* have been identified as associated with neuroimaging measurements, such as brain morphology, subcortical volume, cortical surface area, and cortical thickness [32, 33].

The second most associated variant from our meta-analysis (rs12885261_T_) exerted eQTL effect on *ARG2* gene, with subjects carrying T allele having higher expression level of the gene, as of QTLbase resource. *ARG2* encodes for an enzyme ubiquitously expressed at low level within the mitochondria, having arginine as substrate. This arginase isoform appears to play important roles in regulation of inflammation and pathogenesis of immune-mediated diseases, thus inducing changes in intracellular levels of arginine, whose metabolism is a critical regulator of innate and adaptive immune responses [34]. A recent study showed the beneficial effect of *ARG2* deletion in suppressing retinal neurodegeneration and inflammation in an experimental model of MS [35]. In another study, there was evidence of a significant reduction of Th17 cells and IL-23 + cells in relevant draining lymph nodes associated with Arg II knockout in murine model [36]. This is in line with our findings, which show that patients carrying T allele, possibly having higher transcriptional level of *ARG2*, also have a higher risk of relapsing and being non-responders to NTZ.

Given the increasing awareness of the importance of molecular interactions in shaping complex traits [37], we then integrated human interactome data with our gene-based association statistics using a module search algorithm, with further investigation of topological properties of nodes/genes. The network-based approach, as a complementary strategy, can enhance understanding of molecular mechanisms: the module detected from overlapping our meta-analyzed statistics onto STRING interactome pointed to multiple enriched GO terms. Among these, we found a significant enrichment of terms semantically related to Wnt/β-catenin signaling. It is known that this pathway plays important roles in oligodendrocyte development and myelin formation [38] and its dysregulation may hamper BBB formation. Once the barrier is fully formed, this pathway is also essential to maintain its properties in the adult CNS. Furthermore, it was found that inducible inhibition of this pathway in endothelial cells resulted in clinically exacerbated EAE, thus suggesting that reactivation of Wnt/β-catenin signaling might be beneficial to limit BBB leakage and immune cell infiltration into the CNS [39].

The network approach could then also highlight key players involved in response to treatment: central genes in the network, which would go undetected due to their milder association level, can in fact gain relevance because of their sharing many functional links with other response-associated genes. We focused our attention on two genes, *GRB2* and *LRP6*, which were topologically relevant nodes within our detected module, while being also associated with response and that already showed prior evidence of association with MS from multiple studies.

The *LRP6* gene (lipoprotein receptor-related protein 6) encodes a transmembrane cell surface protein. It plays a key role in the Wnt/β-catenin signaling pathway, being a member of the transmembrane receptor complex to which the Wnt ligand binds, allowing cytosolic β-catenin accumulation and translocation to the nucleus through transcription and regulations of target genes. β-catenin mediates negative effect on differentiation of oligodendrocytes progenitor cells, thus affecting the process of myelin sheath formation: this was confirmed by experimental studies in which expression levels of *LRP6* were markedly increased in RRMS patients and in cuprizone-induced demyelination mice [40, 41]. Furthermore, additional evidence inferred from murine model indicates that enhanced β-catenin expression in T cells leads to aberrant and Th1-biased T-cell activation, infiltration of activated T cells into the spinal cord, and enhanced expression of integrin α4β1 through regulation of *Itgb1* and *Itga4* genes that encode for α4β1/VLA-4 subunits β1 and α4, α4β1/VLA-4 being one of the main two targets of Natalizumab, preventing migration of autoreactive leukocytes through the blood-brain barrier and preventing inflammation [7, 42].

The *GRB2* gene, which encodes for the growth factor receptor bound protein, was found associated in the gene-based meta-analysis and detected as a central gene in the top-ranking module (top 5% percentile in betweenness). The gene is ubiquitously expressed and encodes an adaptor protein, which facilitates the formation of complexes to integrate signals from a wide array of binding partners to inner signaling pathways [43].

The gene acts as a modifier of Wnt/β-catenin signaling, synergizing with multiple components of this pathway, including *LRP6,* to amplify β-catenin dependent transcription. Both in silico and in vivo evidence demonstrate that *GRB2* operates either downstream of, or in parallel with, β-catenin to drive LEF/TCF-mediated transcription of specific genes, including *ITGB1* and *ITGA4*. *GRB2* itself acts downstream of external growth factor receptors and integrins thus providing a way for cells to fine-tune Wnt/β-catenin signaling depending on the extracellular context [44]. Moreover, in mouse, *Grb2*-deficient T cells are impaired in their development and maturation and were found to favor the induction of EAE [45].

Notably, the gene has already been reported as one of the most topologically relevant genes in another network-based study, which jointly investigated two MS GWAS susceptibility cohorts [46]. Further, the intronic variant rs9900529 in *GRB2* was one of the 200 non-MHC loci identified in the to date largest multi-centric study of MS genetic risk from the IMSGC [2] and it has been identified as associated with the response to interferon-beta in MS [47].

More generally, we did not identify significant association of the 200 non-MHC loci from IMSGC study of susceptibility, after Bonferroni correction [2], nor of the genome-wide significant variant rs10191329 in the *DYSF–ZNF638* locus, emerged from the IMSGC study on progression [12] (data not shown).

There are some limitations that must be acknowledged regarding our study. The first concerns the fact that it is under-powered for a genome-wide scan. This is particularly true for the two smaller Italian and German cohort, for which effect estimates exhibited as expected high standard errors with wide confidence intervals. This of course impacted in the fixed-effects meta-analysis, in which contribution of estimates from the two smaller cohorts was down-weighted given their lower precision.

We sought to partially mitigate this issue by complementing GWAS with pathway and network level of analysis. In doing so, given the importance of regulatory information demonstrated by the enrichment of GWAS signals in eQTL loci [48], we also tried to boost signals integrating with SNP–gene assignment information derived from robustly established eQTLs and sQTLs from tissues that are relevant for MS.

Another limitation, which is typical of network-based studies, refers to the fragmented interactome information, since the current knowledge of protein and gene interactions is incomplete and static. We decided, however, to only retain high-confidence links, drawn from the most reliable sources of evidence of STRING repository, such as PPI, co-expression, and functional databases.

Finally, we are aware of the limited sensitivity of relapses, compared to MRI parameters, for the assessment of response to Natalizumab. We considered relapses as clinical outcome of response to maximize the number of patients that could be included in the study. To increase the chance for detecting clinical relapses, we used a period of observation up to 4 years, to obtain data on a medium-term follow-up.

In conclusion, by investigating a multi-centric cohort of MS patients treated with NTZ, we were able to highlight a variant with a putative role in response to drug, rs11132400, and two genes already implicated in MS pathogenesis, *GRB2* and *LRP6*. In addition, from the network module perspective, we report an enrichment of Wnt/β-catenin signaling pathway, which is an essential component for BBB formation and maintenance. A replication study of these findings in an independent cohort would be desirable to support future clinical applications.

## Supplementary Information

Below is the link to the electronic supplementary material.

Supplementary file1 (PDF 280 KB)

Supplementary file2 (PDF 161 KB)

Supplementary file3 (XLS 17 KB)

Supplementary file4 (XLS 13 KB)

Supplementary file5 (XLS 163 KB)

Supplementary file6 (XLS 12 KB)

Supplementary file7 (XLS 10 KB)

Supplementary file8 (XLS 25 KB)
