# Correction: Pharmacogenomics of clinical response to Natalizumab in multiple sclerosis: a genome-wide multi-centric association study

**DOI:** 10.1007/s00415-025-13108-x

**Published:** 2025-05-27

**Authors:** Ferdinando Clarelli, Andrea Corona, Kimmo Pääkkönen, Melissa Sorosina, Alen Zollo, Fredrik Piehl, Tomas Olsson, Pernilla Stridh, Maja Jagodic, Bernhard Hemmer, Christiane Gasperi, Adil Harroud, Klementy Shchetynsky, Alessandra Mingione, Elisabetta Mascia, Kaalindi Misra, Antonino Giordano, Maria Laura Terzi Mazzieri, Alberto Priori, Janna Saarela, Ingrid Kockum, Massimo Filippi, Federica Esposito, Filippo Martinelli Boneschi

**Affiliations:** 1 Laboratory of Human Genetics of Neurological Disorders, IRCCS San Raffaele Scientific Institute, Via Olgettina 60, Milan, Italy; 2 Laboratory of Precision Medicine of Neurological Diseases, Department of Health Science, University of Milan, Milan, Italy; 3 CRC “Aldo Ravelli” for Experimental Brain Therapeutics, Department of Health Science, University of Milan, Milan, Italy; 4 Institute for Molecular Medicine Finland (FIMM), University of FI Helsinki, Helsinki, Finland; 5 The Karolinska Neuroimmunology & Multiple Sclerosis Centre, Department of Clinical Neuroscience, Karolinska Institutet, Center for Molecular Medicine, Karolinska University Hospital, Visionsgatan 18, 171 76 Stockholm, Sweden; 6 Department of Neurology, School of Medicine, Technical University of Munich, Klinikum Rechts Der Isar, Ismaninger Str. 22, Munich, Germany; 7 Munich Cluster for Systems Neurology (SyNergy), Munich, Germany; 8 Department of Neurology and Neurosurgery, McGill University, Montréal, QC Canada; 9 Neurology Unit, IRCCS San Raffaele Scientific Institute, Via Olgettina 60, 20132 Milan, Italy; 10 Clinical Neurology Unit, Azienda Socio-Sanitaria Territoriale Santi Paolo E Carlo and Department of Health Sciences, University of Milan, Milan, Italy; 11 Neurorehabilitation Unit, IRCCS San Raffaele Scientific Institute, Via Olgettina 48, Milan, Italy; 12 Neurophysiology Service, IRCCS San Raffaele Scientific Institute, Via Olgettina 60, Milan, Italy; 13 Neuroimaging Research Unit, Division of Neuroscience, IRCCS San Raffaele Scientific Institute, Via Olgettina 60, Milan, Italy; 14 Vita-Salute San Raffaele University, Via Olgettina, 60, Milan, Italy

**Correction: Journal of Neurology (2024) 271:7250–7263** 10.1007/s00415-024-12608-6

In the original version of this article, author’s name Filippo Martinelli Boneschi was incorrectly written as Filippo Giovanni Martinelli Boneschi.

In Table 2, first two columns were missing. The Table 2 which previously appeared as

**Table 2:** List of top-associated variants after meta-analysis. The list is obtained upon a clumping procedure (see *Methods* for details), filtered at *p* < 5 × 10^–5^. The effect allele is the minor allele

Position	Minor allele	Major allele	MAF	P	OR	SE	*I*^2^	Gene	Genomic context	Distance to gene
187,410,850	T	C	0.268	1.328E−06	0.582	0.112	0	*F11-AS1*	ncRNA_intronic	
68,071,570	T	C	0.421	1.678E−06	1.538	0.090	0	*PIGH; ARG2*	Intergenic	Dist = 4552; Dist = 15,067
111,037,829	T	A	0.251	2.793E−06	0.596	0.111	13	*KLF4; ACTL7B*	Intergenic	Dist = 785,779; Dist = 579,040
20,863,249	G	A	0.057	4.804E−06	2.122	0.165	0	*GJB6; CRYL1*	Intergenic	Dist = 56,715; Dist = 114,557
98,275,102	C	G	0.429	6.938E−06	0.650	0.096	34	*LOC101927310; LINC00923*	Intergenic	Dist = 171,174; Dist = 10,744
47,775,385	A	G	0.230	1.187E−05	1.561	0.102	0	*SEMA6D*	Intronic	
21,728,917	A	G	0.142	1.191E−05	0.510	0.154	0	*ZNF385D*	Intronic	
187,402,360	A	G	0.480	1.233E−05	1.496	0.092	0	*F11-AS1*	ncRNA_intronic	
115,392,100	A	G	0.347	1.259E−05	1.476	0.089	0	*GAP43*	Intronic	
12,783,519	T	C	0.075	1.325E−05	1.934	0.151	0	*LINC00681; TRMT9B*	Intergenic	Dist = 107,719; Dist = 19,663
43,181,587	T	C	0.080	1.362E−05	1.918	0.150	0	*A4GALT; ARFGAP3*	Intergenic	Dist = 64,280; Dist = 10,922
79,303,163	G	A	0.430	1.475E−05	1.469	0.089	0	*WWOX; MAF*	Intergenic	Dist = 56,599; Dist = 324,572
98,347,870	C	G	0.429	1.494E−05	0.676	0.091	57	*LINC00923*	ncRNA_intronic	
27,204,531	C	T	0.095	2.087E−05	1.775	0.135	42	*ABI1; FAM238C*	Intergenic	Dist = 54,515; Dist = 15,604
47,746,678	C	G	0.075	2.692E−05	1.839	0.145	29	*STIL*	Exonic	
113,293,608	G	A	0.411	2.744E−05	1.461	0.090	0	*TUBGCP3; ATP11AUN*	Intergenic	Dist = 51,109; Dist = 7750
606,173	C	A	0.143	2.862E−05	1.646	0.119	0	*B4GALNT3*	Intronic	
83,766,280	A	C	0.114	2.909E−05	1.739	0.132	48	*DLG2*	Intronic	
236,647,736	T	C	0.233	2.939E−05	1.495	0.096	0	*EDARADD*	UTR3	NM_145861:c.*1787C>T; NM_080738:c.*1787C>T
93,450,212	A	G	0.295	3.023E−05	1.471	0.093	0	*RUNX1T1; LOC102724710*	Intergenic	Dist = 334,599; Dist = 127,459
4,314,917	A	G	0.220	3.087E−05	1.523	0.101	0	*CSMD1*	Intronic	
81,279,746	A	G	0.271	3.116E−05	1.479	0.094	6.4	*SPRY2; LINC00377*	Intergenic	Dist = 364,485; Dist = 312,780
111,023,899	T	C	0.404	3.369E−05	1.441	0.088	0	*KLF4; ACTL7B*	Intergenic	Dist = 771,849; Dist = 592,970
189,243,943	T	C	0.413	3.424E−05	1.433	0.087	0	*TPRG1; TP63*	Intergenic	Dist = 200,850; Dist = 70,592
114,813,313	C	T	0.267	3.459E−05	1.483	0.095	0	*TBX5*	Intronic	
110,446,839	G	A	0.413	3.508E−05	1.433	0.087	0	*IRS2; LINC00396*	Intergenic	Dist = 7909; Dist = 258,793
177,987,660	G	A	0.137	3.639E−05	1.617	0.116	0	*CRYZL2P*	ncRNA_intronic	
31,271,788	A	G	0.072	3.733E−05	1.845	0.148	60	*OVOS2*	ncRNA_intronic	
206,468,850	T	G	0.419	4.007E−05	1.433	0.088	0	*PARD3B*	Intronic	
54,692,961	G	A	0.116	4.025E−05	1.652	0.122	6.9	*TINAG; FAM83B*	Intergenic	Dist = 438,021; Dist = 18,608
141,938,633	C	T	0.069	4.217E−05	1.890	0.155	74	*MGAM2; MOXD2P*	Intergenic	Dist = 16,509; Dist = 1923
81,959,114	T	C	0.499	4.222E−05	0.697	0.088	5.8	*ATP6AP1L; MIR3977*	Intergenic	Dist = 343,363; Dist = 176,860
158,058,545	T	C	0.436	4.257E−05	1.455	0.092	0	*ZDHHC14*	Intronic	
180,648,923	C	T	0.480	4.571E−05	0.699	0.088	0	*LINC01098; LINC00290*	Intergenic	Dist = 1,737,019; Dist = 1,336,320
83,182,354	A	G	0.299	4.605E−05	1.458	0.093	0	*DLG2*	Intronic	
10,732,844	G	A	0.050	4.841E−05	2.053	0.177	53	*TEKT5*	Intronic	
130,499,623	G	A	0.096	4.929E−05	1.749	0.138	42	*LINC01163; LINC02667*	Intergenic	Dist = 383,633; Dist = 211,474
156,675,608	A	C	0.135	4.934E−05	1.642	0.122	0	*GUCY1A1; GUCY1B1*	Intergenic	Dist = 17,399; Dist = 4565
50,679,081	T	G	0.372	4.966E−05	1.448	0.091	0	*DEFB112; TFAP2D*	Intergenic	Dist = 661,439; Dist = 2158

For each variant, the gene harboring it or nearest gene(s) are reported, together with its genomic context and distance in base-pairs from the nearest genes, as of ANNOVAR annotation

Abbreviations: MAF (Minor Allele Frequency), P (p value from fixed-effect meta-analysis), OR (odds ratio from fixed-effects meta-analysis), SE (standard error), I^2^ (heterogeneity index)

 but Table 2 should have appeared as shown below.

**Table 2:** List of top associated variants after meta-analysis

SNP	Chromosome	Position	Minor allele	Major allele	MAF	P	OR	SE	*I*^2^	Gene	Genomic context	Distance to gene
rs11132400	4	187410850	T	C	0.268	1.328E−06	0.582	0.112	0	*F11-AS1*	ncRNA_intronic	
rs12885261	14	68071570	T	C	0.421	1.678E−06	1.538	0.090	0	*PIGH; ARG2*	Intergenic	dist = 4552; dist = 15,067
rs1323374	9	111037829	T	A	0.251	2.793E−06	0.596	0.111	13	*KLF4; ACTL7B*	Intergenic	dist = 785,779; dist = 579,040
rs61166479	13	20863249	G	A	0.057	4.804E−06	2.122	0.165	0	*GJB6; CRYL1*	Intergenic	dist = 56,715; dist = 114,557
rs1032954	15	98275102	C	G	0.429	6.938E−06	0.650	0.096	34	*LOC101927310; LINC00923*	Intergenic	dist = 171,174; dist = 10,744
rs75772884	15	47775385	A	G	0.230	1.187E−05	1.561	0.102	0	*SEMA6D*	Intronic	
rs34736466	3	21728917	A	G	0.142	1.191E−05	0.510	0.154	0	*ZNF385D*	Intronic	
rs12649886	4	187402360	A	G	0.480	1.233E−05	1.496	0.092	0	*F11-AS1*	ncRNA_intronic	
rs13073759	3	115392100	A	G	0.347	1.259E−05	1.476	0.089	0	*GAP43*	Intronic	
rs2977141	8	12783519	T	C	0.075	1.325E−05	1.934	0.151	0	*LINC00681; TRMT9B*	Intergenic	dist = 107,719; dist = 19,663
rs7286822	22	43181587	T	C	0.080	1.362E−05	1.918	0.150	0	*A4GALT; ARFGAP3*	Intergenic	dist = 64,280; dist = 10,922
rs9922330	16	79303163	G	A	0.430	1.475E−05	1.469	0.089	0	*WWOX; MAF*	Intergenic	dist = 56,599; dist = 324,572
rs72752604	15	98347870	C	G	0.429	1.494E−05	0.676	0.091	57	*LINC00923*	ncRNA_intronic	
rs1815323	10	27204531	C	T	0.095	2.087E−05	1.775	0.135	42	*ABI1; FAM238C*	Intergenic	dist = 54,515; dist = 15,604
rs10789505	1	47746678	C	G	0.075	2.692E−05	1.839	0.145	29	*STIL*	Exonic	
rs9577824	13	113293608	G	A	0.411	2.744E−05	1.461	0.090	0	*TUBGCP3; ATP11AUN*	Intergenic	dist = 51,109; dist = 7750
rs2159600	12	606173	C	A	0.143	2.862E−05	1.646	0.119	0	*B4GALNT3*	Intronic	
rs12806160	11	83766280	A	C	0.114	2.909E−05	1.739	0.132	48	*DLG2*	Intronic	
rs6428955	1	236647736	T	C	0.233	2.939E−05	1.495	0.096	0	*EDARADD*	UTR3	NM_145861:c.*1787C > T; NM_080738:c.*1787C > T
rs1072934	8	93450212	A	G	0.295	3.023E−05	1.471	0.093	0	*RUNX1T1; LOC102724710*	Intergenic	dist = 334,599; dist = 127,459
rs1217680	8	4314917	A	G	0.220	3.087E−05	1.523	0.101	0	*CSMD1*	Intronic	
rs12428095	13	81279746	A	G	0.271	3.116E−05	1.479	0.094	6.4	*SPRY2; LINC00377*	Intergenic	dist = 364,485; dist = 312,780
rs10739255	9	111023899	T	C	0.404	3.369E−05	1.441	0.088	0	*KLF4; ACTL7B*	Intergenic	dist = 771,849; dist = 592,970
rs6803844	3	189243943	T	C	0.413	3.424E−05	1.433	0.087	0	*TPRG1; TP63*	Intergenic	dist = 200,850; dist = 70,592
rs7960396	12	114813313	C	T	0.267	3.459E−05	1.483	0.095	0	*TBX5*	Intronic	
rs1414319	13	110446839	G	A	0.413	3.508E−05	1.433	0.087	0	*IRS2; LINC00396*	Intergenic	dist = 7909; dist = 258,793
rs7537568	1	177987660	G	A	0.137	3.639E−05	1.617	0.116	0	*CRYZL2P*	ncRNA_intronic	
rs112417083	12	31271788	A	G	0.072	3.733E−05	1.845	0.148	60	*OVOS2*	ncRNA_intronic	
rs13031793	2	206468850	T	G	0.419	4.007E−05	1.433	0.088	0	*PARD3B*	Intronic	
rs9475026	6	54692961	G	A	0.116	4.025E−05	1.652	0.122	6.9	*TINAG; FAM83B*	Intergenic	dist = 438,021; dist = 18,608
rs59801030	7	141938633	C	T	0.069	4.217E−05	1.890	0.155	74	*MGAM2; MOXD2P*	Intergenic	dist = 16,509; dist = 1923
rs10040233	5	81959114	T	C	0.499	4.222E−05	0.697	0.088	5.8	*ATP6AP1L; MIR3977*	Intergenic	dist = 343,363; dist = 176,860
rs618823	6	158058545	T	C	0.436	4.257E−05	1.455	0.092	0	*ZDHHC14*	Intronic	
rs4444875	4	180648923	C	T	0.480	4.571E−05	0.699	0.088	0	*LINC01098; LINC00290*	Intergenic	dist = 1,737,019; dist = 1,336,320
rs2068076	11	83182354	A	G	0.299	4.605E−05	1.458	0.093	0	*DLG2*	Intronic	
rs76674464	16	10732844	G	A	0.050	4.841E−05	2.053	0.177	53	*TEKT5*	Intronic	
rs12265034	10	130499623	G	A	0.096	4.929E−05	1.749	0.138	42	*LINC01163; LINC02667*	Intergenic	dist = 383,633; dist = 211,474
rs6838876	4	156675608	A	C	0.135	4.934E−05	1.642	0.122	0	*GUCY1A1; GUCY1B1*	Intergenic	dist = 17,399; dist = 4565
rs1923523	6	50679081	T	G	0.372	4.966E−05	1.448	0.091	0	*DEFB112; TFAP2D*	Intergenic	dist = 661,439; dist = 2158

The list is obtained upon a clumping procedure (see Methods for details), filtered at *p* < 5 × 10^−5^. The effect allele is the minor allele

For each variant, the gene harboring it or nearest gene(s) are reported, together with its genomic context and distance in base-pairs from the nearest genes, as of ANNOVAR annotation

MAF (Minor Allele Frequency), P (p-value from fixed-effect meta-analysis), OR (odds ratio from fixed-effects meta-analysis), SE (standard error), I^2^ (heterogeneity index)

