# Supplementary figures and images for: Pharmacogenomics of clinical response to Natalizumab in multiple sclerosis: a genome-wide multi-centric association study
Source: J Neurol. 2024 Sep 12;271(11):7250–63. doi: 10.1007/s00415-024-12608-6 (PMC11561017; doi:10.1007/s00415-024-12608-6)

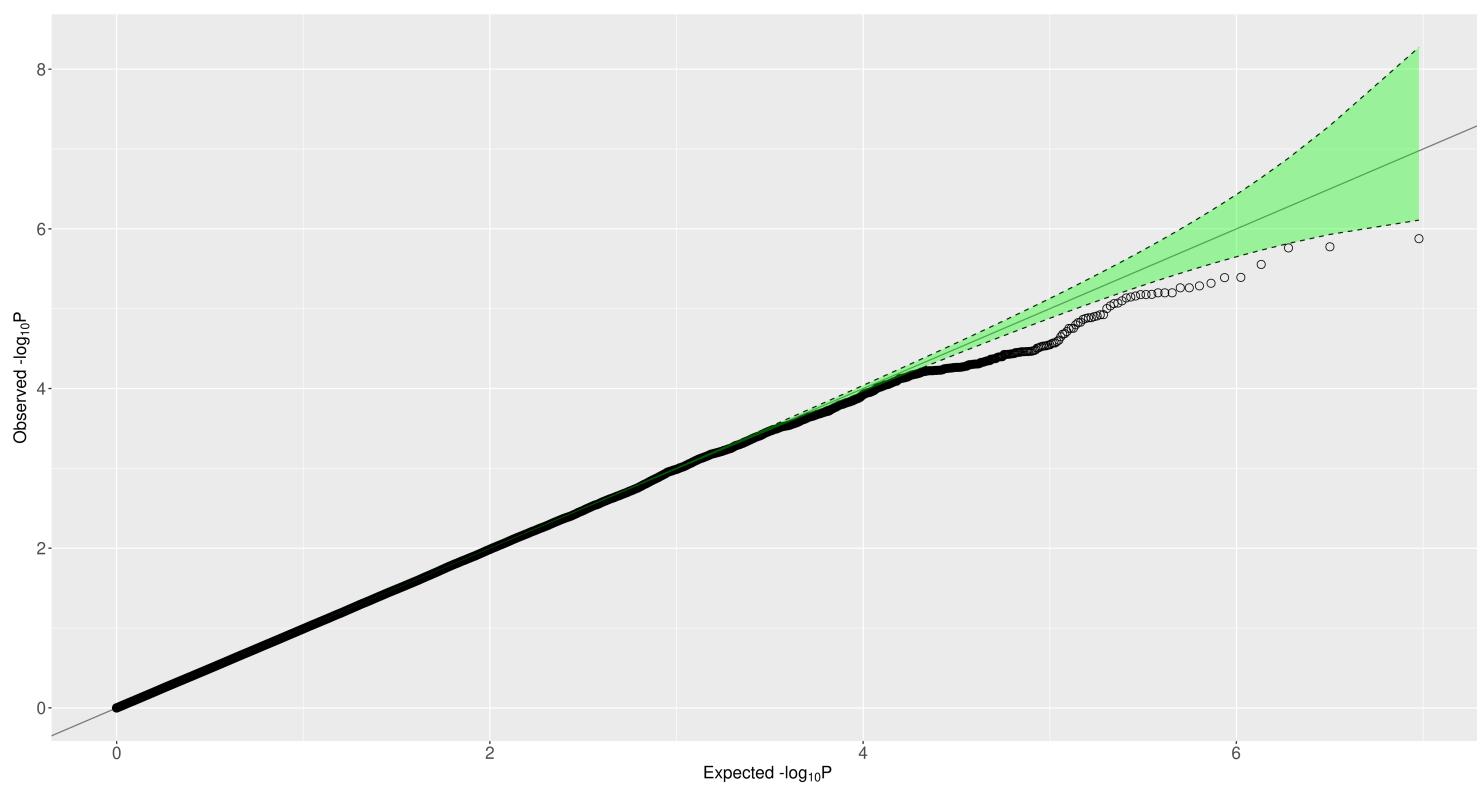

Supplement: Supplementary file 1 — Supplementary file1 (PDF 280 KB) [file 415_2024_12608_MOESM1_ESM.pdf]

a

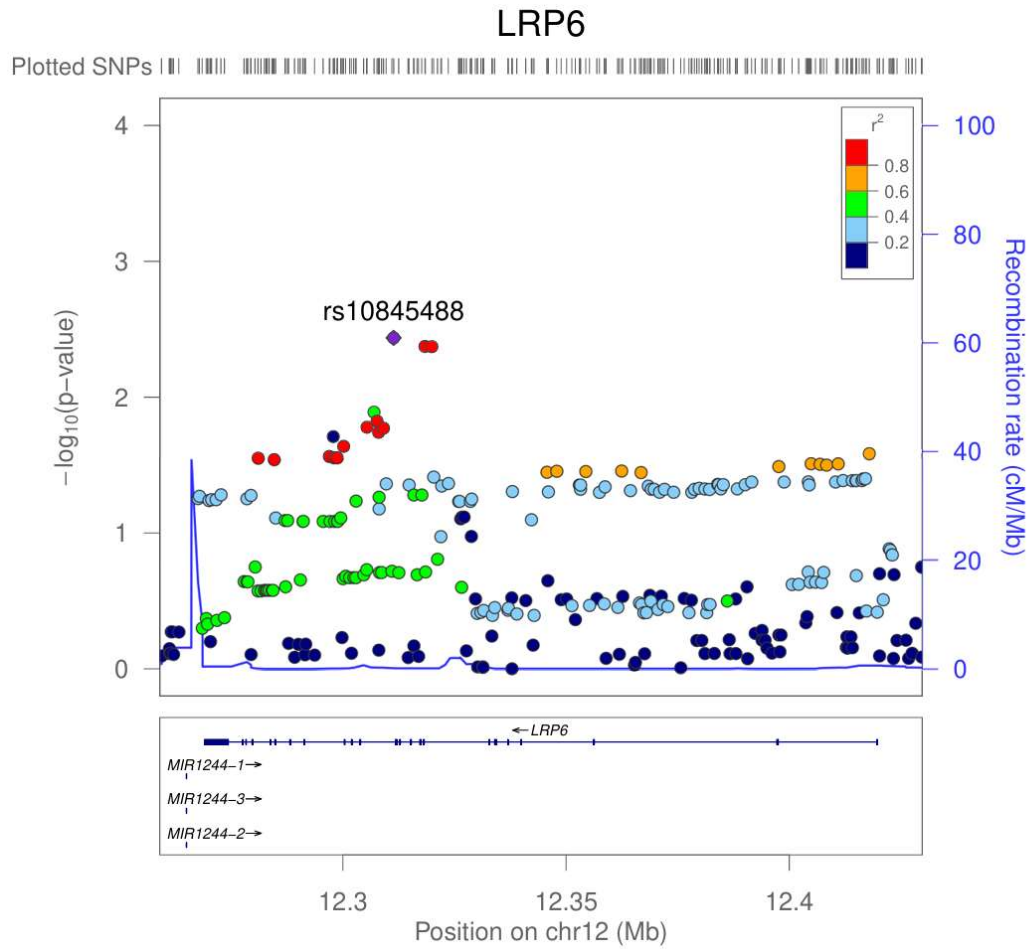

b

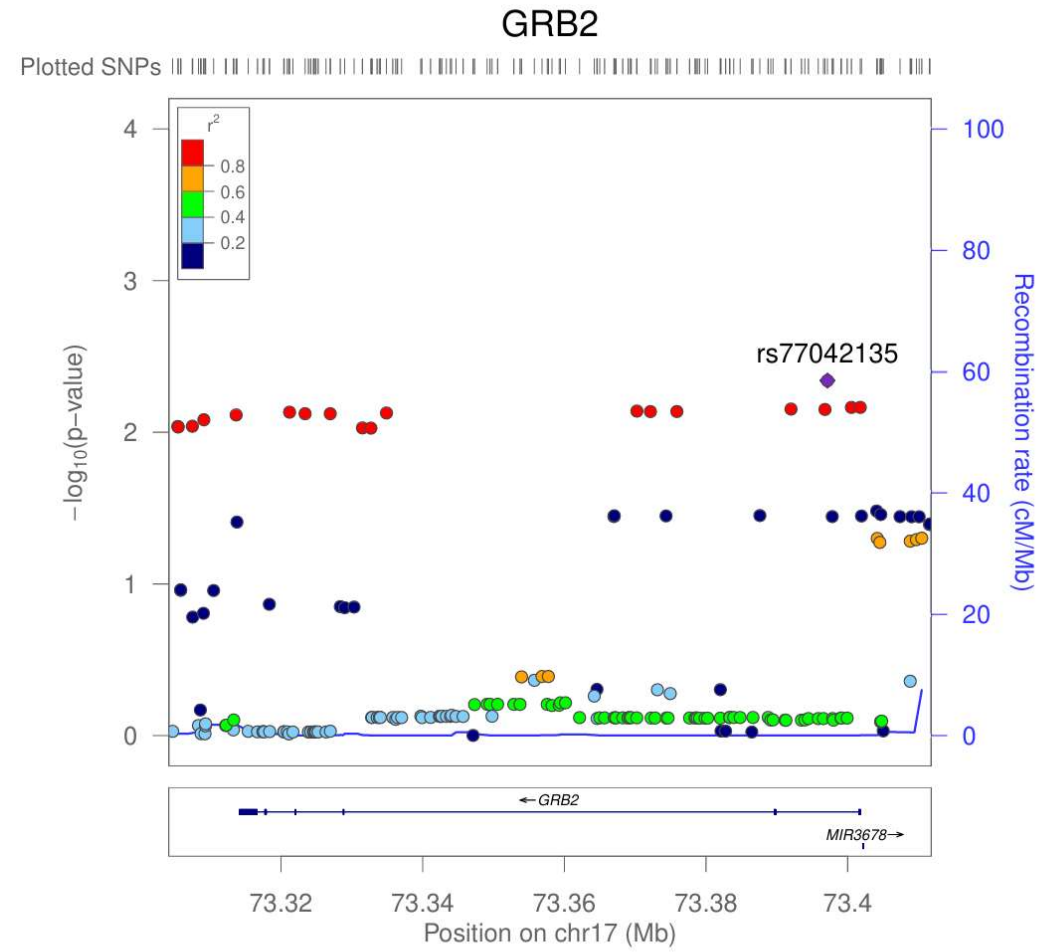

Supplement: Supplementary file 2 — Supplementary file2 (PDF 161 KB) [file 415_2024_12608_MOESM2_ESM.pdf]
